# Prediction of depressive symptoms in middle-aged and older adult hospitalized patients with chronic diseases: a multicenter study in Anhui, China using machine learning methods

**DOI:** 10.3389/fpubh.2026.1865279

**Published:** 2026-07-09

**Authors:** Shouqiang Huang, Huan Liu, Qingwei Liu, Xinyu Hu, Xu Qin, Guangliang Mei, Xiaoli Wang, Shijia Gu, Rui Hong, Lingling Pan, Ming Zhang, Mingfen Tao

**Affiliations:** 1 Department of Ophthalmology, Wuhu Second People's Hospital, Wuhu, Anhui, China; 2 Department of Hemodialysis, The First Affiliated Yijishan Hospital of Wannan Medical University (Yijishan Hospital of Wannan Medical University), Wuhu, Anhui, China; 3 Department of Nursing, Shandong Provincial Hospital Affiliated to Shandong First Medical University (Shandong Provincial Hospital), Jinan, Shandong, China; 4 Department of Cardiovascular, The Second Affiliated Hospital of Wangnan Medical College, Wuhu, Anhui, China; 5 Department of Interventional, The First Affiliated Hospital of Wannan Medical University (Yijishan Hospital of Wannan Medical University), Wuhu, Anhui, China; 6 The First Affiliated Hospital of Wannan Medical University (Yijishan Hospital of Wannan Medical University), Wuhu, China; 7 China Hospital Reform and Development Research Institute of Nanjing University, Nanjing Drum Tower Hospital, Nanjing, China; 8 Department of General Practice, The First Affiliated Yijishan Hospital of Wannan Medical University (Yijishan Hospital of Wannan Medical University), Wuhu, Anhui, China; 9 Department of Graduate School, Wanna Medical University, Wuhu, Anhui, China; 10 Department of Nursing, The First Affiliated Yijishan Hospital of Wannan Medical University (Yijishan Hospital of Wannan Medical University), Wuhu, Anhui, China; 11 Department of Cardiology, The First Affiliated Yijishan Hospital of Wannan Medical University (Yijishan Hospital of Wannan Medical University), Wuhu, Anhui, China; 12 School of Innovation and Entrepreneurship, Wanna Medical University, Wuhu, Anhui, China; 13 Key Laboratory of Philosophy and Social Science of Anhui Province on Adolescent Mental Health and Crisis Intelligence Intervention, Hefei Normal University, Hefei, China

**Keywords:** China, chronic diseases, depressive symptoms, hospitalized, machine learning

## Abstract

**Objective:**

This cross-sectional study has dual objectives: to investigate the predictive value of machine learning (ML) for the prevalence of depressive symptoms in middle-aged and older adult hospitalized patients with chronic diseases, and to identify significant factors influencing depressive symptoms in this population.

**Methods:**

A cross-sectional study was conducted among 618 hospitalized middle-aged and older adult patients with chronic diseases. Participants completed questionnaires assessing depression, chronic illness stigma, oral frailty, social isolation, family health, and demographic characteristics. The XG Boost model algorithm was employed for feature selection and variable importance ranking. A predictive model was constructed to assess the risk of depressive symptoms, and the feature importance honeycomb plot was utilized to illustrate the relationships between variables and prediction outcomes.

**Results:**

The study found multiple risk factors significantly associated with depression, including gender, place of residence, number of surgeries in the past year, hospitalization in the past 2 years, social isolation, level of education, comorbidity, age, malignant disease, oral frailty, stigma associated with illness, and family health. The XG Boost model demonstrated optimal predictive performance, achieving an AUC value of 0.931. Key predictive factors included stigma scale for chronic, family health, oral frailty, social isolation, malignant disease, and hospitalization in the past 2 years.

**Conclusion:**

This study constructed a risk prediction model for depressive symptoms in middle-aged and older adult hospitalized patients with chronic diseases, providing an important reference basis for mental health interventions in this population.

## Introduction

According to China’s seventh national population census, there are currently 264 million people aged 60 and above in China, accounting for 18.7% of the total population (1). According to the “National Assessment Report on Aging and Health in China” released by the World Health Organization (WHO), it is projected that by 2030, the prevalence of chronic non-communicable diseases among the older adults in China will increase by at least 40%, with over 75% of the older adult population facing multimorbidity issues (2). It is reported that approximately 62.8% of Chinese residents aged 45 and above suffer from at least one non-communicable disease, with a higher prevalence of multimorbidity among the older adult population (3).

While focusing on the impact of chronic diseases on the physical health of the middle-aged and older adults, attention should also be paid to their harm to mental health. After contracting chronic diseases, individuals need to face both financial pressure and psychological distress, which can easily lead to an increased risk of depression (4). Particularly for hospitalized older adult patients with chronic diseases, it is necessary to investigate the current status and influencing factors of their depressive symptoms, and explore corresponding relief mechanisms. Depression is one of the most common mental health issues. The World Health Organization estimates that approximately 350 million people worldwide suffer from depression (5, 6). Foreign studies have shown (7) that the overall incidence of depression in the older adult ranges from 10.80 to 56.90%, while domestic research (8) indicates that the prevalence of depression among Chinese adults aged 60 and above is between 11 and 57%.

Chronic diseases are characterized by prolonged duration, insidious onset, and the inability to be cured but only managed. They impose a heavy burden on patients and their families. The deterioration of the condition can lead to emotional numbness and pessimism, triggering a series of complex psychological reactions—tension, anxiety, pessimism, despair, anger, irritability, and fear. Additionally, reduced communication with relatives and friends and decreased social activities can exacerbate the generation of negative emotions (9). Relevant studies have found that patients who are older, have longer durations of chronic diseases, suffer from more types of chronic conditions, and have poorer self-care abilities are more likely to adopt negative coping strategies toward their illnesses, leading to depressive mood disorders or even severe organic mental disorders, which in turn exacerbate their physical conditions (10). The study conducted by Zhu CS et al. based on large-scale population data revealed that there was an association between depressive symptoms and chronic diseases among middle-aged and older adult Chinese individuals compared to the general population, with over 50% of those exhibiting depressive symptoms suffering from at least one chronic disease (11). Research indicates that the coexistence of depression and chronic conditions exacerbates adverse outcomes, including accelerated functional decline, poorer disease management, and increased mortality risk (12). Older adult individuals with depressive symptoms report lower life satisfaction, impaired activities of daily living (ADLs), and heightened vulnerability to elder abuse, which in turn predicts a twofold increase in depressive symptoms (13, 14). Therefore, paying attention to the mental health of middle-aged and older adult patients with chronic diseases, and strengthening early screening, prevention, and early intervention for the comorbidity of chronic diseases and depressive symptoms in the middle-aged and older adults, can help reduce the harm caused by the comorbidity of chronic diseases and depressive symptoms in the middle-aged and older adults, improve the mental health status of the middle-aged and older adult patient population with chronic diseases, and promote healthy aging.

In China, the proportion of middle - aged and older adult patients with multiple chronic diseases who exhibit depressive symptoms is as high as 45.65% (15). Depressive symptoms in middle-aged and older adult patients with chronic diseases are influenced by various risk factors including biological, psychological, and social factors. Studies have found that 54% of chronic disease patients report greater impairment from psychological symptoms than from the physical symptoms of their primary condition. Such psycho physiological interactions can lead to decreased treatment adherence, increased symptom burden, and heightened suicidal ideation, trapping patients in a vicious cycle of “physical-psychological-social functioning” triple impairment (16). Therefore, focusing on the mental health of older adult patients, particularly depression comorbid with chronic diseases, has become a crucial component in improving the overall health status of the middle-aged and older adult population.

Currently, there is limited research on depressive symptoms in middle-aged and older adult hospitalized patients with chronic diseases. Researchers have identified several risk factors for depressive symptoms in this population, including disease-related factors, functional status, physiological factors, and coping mechanisms (17). Yu (18) reported in her study that the incidence of depression in older adult patients with chronic diseases was 54.67%, and there was a close relationship between somatization symptoms and depressive mood. Chun et al. (19) found in their research that approximately 40.0% of older adult hospitalized patients exhibited depressive symptoms, with family support, functional independence, and widowhood being independent influencing factors for depressive symptoms. Therefore, this study investigates middle-aged and older adult hospitalized patients with chronic diseases to assess their depression status and related influencing factors. It aims to provide valid data for improving life prognosis, reducing readmission rates, alleviating family and socioeconomic burdens, and enhancing patient compliance among middle-aged and older adult chronic disease patients. The findings will serve as a reference for developing scientific and feasible intervention strategies to improve the mental health status of middle-aged and older adult patients with chronic conditions.

Oral frailty refers to a new conceptual understanding related to aging, highlighting a progressive deterioration of oral function that coincides with a decrease in both cognitive and physical abilities, which includes neurodegeneration associated with Alzheimer’s disease as well as alterations in oral microbiota (20). In a meta-analysis, researchers Cademartori et al. (21) found a positive correlation between depressive symptoms and the risk of oral diseases (such as dental caries, periodontal disease, and tooth loss). However, the correlation between depressive symptoms and oral frailty in middle-aged and older adult patients with chronic diseases remains poorly understood. Hence, it is essential to consider the correlation between oral health and depressive symptoms in middle-aged and older adult hospitalized patients with chronic diseases.

Family health can be defined as “a resource within the family unit that arises from the convergence of the health statuses of individual family members, their interactions and abilities, in addition to the family’s available physical, social, emotional, economic, and medical resources” (22). This concept integrates core elements such as family structure, family functioning, and family social networks, aiming to enhance the family’s ability to access external resources and social support, while emphasizing health-related factors that connect individual health with societal health (23). Therefore, family health may become an important target for individual health interventions in the future. Studies indicate that robust family health resources, including emotional cohesion and healthy lifestyle practices, act as protective factors against depressive symptoms in middle-aged and older adults (24). Conversely, poor family health—marked by dysfunction in roles, problem-solving, or emotional processes—exacerbates depressive symptoms, particularly in vulnerable groups such as pregnant women, middle-aged and older adult, and migrant populations (25, 26).

Stigma denotes unfavorable or mistaken stereotypes concerning a particular group of individuals and arises from “poorly justified knowledge structures that lead to discrimination” (27). Individuals with illness-related stigma experience negative emotional states such as discrimination, self-blame, and shame due to being labeled by their disease. These experiences have severe adverse effects on their quality of life, treatment adherence, and self-efficacy (28). Studies have indicated that chronic disease patients exhibit illness-related stigma, which is closely associated with psychological disorders like anxiety and depression (29).

Machine learning (ML) models, with their powerful data processing capabilities and distinct advantages in building predictive models, are widely applied in constructing psychological models, which may help identify depressive symptoms in middle-aged and older adult patients with chronic diseases. Although several recent studies have applied machine learning approaches to predict depressive symptoms among Chinese middle-aged and older adults, most have relied on large-scale, community-based cohorts such as the China Health and Retirement Longitudinal Study (CHARLS) or the Chinese Longitudinal Healthy Longevity Survey (CLHLS) (30–33). For instance, Xia et al. (30) developed a depression risk prediction model for healthy community-dwelling middle-aged and older adults using CHARLS data, while Song et al. (31) constructed machine learning models for older adults in a cross-sectional community sample. Xu et al. (33) focused on older adults with subjective cognitive decline, also from a longitudinal community cohort, and Long et al. (34) employed deep learning methods on CHARLS with external validation in CLHLS. More recently, Wang et al. (35) built an explainable machine learning model for depressive symptoms in community-dwelling older Chinese adults with chronic diseases. Despite these valuable contributions, two critical gaps remain. First, these existing models are derived from nationally representative or community-based samples, which typically include relatively healthy individuals and may not generalize to hospitalized patients with chronic diseases – a population carrying a substantially higher psychological burden. Second, while these studies considered conventional risk factors (e.g., demographics, socioeconomic status, comorbidities, physical function), none have incorporated domain-specific predictors particularly relevant to hospitalized populations, such as oral frailty or disease-related stigma. Our study therefore addresses these gaps by focusing on a real-world clinical cohort of middle-aged and older adult in patients with chronic diseases in China, and by integrating novel predictors (oral frailty and a validated stigma scale) into a machine learning framework. This allows us to examine whether these clinically accessible features improve prediction accuracy in a high-risk, hospitalized setting – an area not covered by previous community-based models (30–35). Therefore, this study aims to (1) detect depressive symptoms in middle-aged and older adult patients with chronic diseases by applying nine machine learning algorithms, and (2) identify predictive factors for depressive symptoms in this population. The findings will provide a reference for reducing negative psychological outcomes and adverse health consequences among middle-aged and older adult chronic disease patients, thereby facilitating their reintegration into society and improving their quality of life.

## Materials and methods

This is a multicenter cross-sectional study. Additionally, the research team strictly adhered to the STROBE (Strengthening the Reporting of Observational Studies in Epidemiology) cross-sectional checklist when preparing the report.

### Participants

In this cross-sectional study, our research team employed a stratified cluster random sampling method to recruit participants from three Grade A tertiary hospitals in Wuhu City, Anhui Province, China, between October 2024 and January 2025. This study recruited 618 middle-aged and older adult patients with chronic diseases from a tertiary Grade A hospital. Inclusion criteria: (a) age≥45 years; (b) meeting the diagnostic criteria for any of the 14 chronic diseases defined in the CHARLS survey (36), including the following conditions: hypertension, dyslipidemia, chronic lung disease, stroke, diabetes or hyperglycemia, heart disease, cancer or malignant tumor, liver disease, kidney disease, gastric or other digestive system diseases, arthritis, rheumatism or asthma; (c) having good language communication skills and clear logical thinking ability; (d) providing informed consent and voluntarily participating in this cross-sectional study. Exclusion criteria: (a) diagnosed with severe mental disorders or memory-related diseases; (b) presence of other serious conditions that may hinder participation in this cross-sectional survey (e.g., severe respiratory failure).

### Measurements

#### Demographic characteristics

To gather socially and demographically pertinent information from participants, a well-organized questionnaire was employed. This instrument was created following an extensive literature review concerning middle-aged and older adult and discussions with pertinent epidemiological specialists. The demographic survey collected data on age, gender, educational attainment, place of residence, employment situation, marital status, living arrangements, and lifestyle habits.

#### Depressive symptoms

The Patient Health Questionnaire (PHQ-9) was used to measure depressive symptoms among middle-aged and older adult patients with chronic diseases over the past 2 weeks. This scale is based on the nine diagnostic criteria for depression from the fourth edition of the Diagnostic and Statistical Manual of Mental Disorders (DSM-IV) published by the American Psychiatric Association in 1994. Each item is scored using a 4-point Likert scale (0 = not at all, 1 = several days, 2 = more than 7 days, 3 = nearly every day), with the total PHQ-9 score ranging from 0 to 27. Higher scores indicate more severe depressive symptoms (37). A total score of 10 or above on the PHQ-9 scale may indicate the presence of depressive symptoms. This scale has been validated in various Chinese populations and has demonstrated good validity and reliability among middle-aged and older adult patients with chronic diseases. The Cronbach’s alpha coefficient of this scale was 0.801 in the present study. We categorized depressive symptoms as a binary variable (presence or absence of depressive symptoms), with a PHQ-9 score ≥10 indicating that participants had depressive symptoms (38).

#### Stigma

In this cross-sectional study, the stigma experienced by participants was assessed using the Chinese version of the Stigma Scale for Chronic Illnesses-8 items (SSCI-8). The SSCI-8 consists of 8 items, with 6 items used to evaluate external stigma and 2 items to assess internal stigma. The scale includes 5 response options: never, rarely, sometimes, often, and always, scored from 1 to 5 points. The total score ranges from 8 to 40 points, with higher scores indicating higher levels of stigma. The Chinese version of the SSCI-8 has demonstrated high reliability and validity, and the scale can be completed in a short period of time. Therefore, the SSCI-8 can be widely applied in clinical settings among patients with chronic illnesses (39).

#### Oral frailty

The oral frailty scale used in this study was developed by Tanaka et al. (40) and was translated into Chinese by Chen Zongmei in 2023 for assessing the oral frailty status of middle-aged and older adult individuals. This scoring scale comprises 8 items, covering 5 dimensions: masticatory ability (2 items), swallowing function (2 items), use of dentures (1 item), social participation (1 item), and oral health-related behaviors (2 items). For items 1–3, a response of “yes” scores 2 points, while a response of “no” scores 0 points. For items 4 and 5, a response of “yes” scores 1 point, while a response of “no” scores 0 points. For items 6–8, a response of “yes” scores 0 points, while a response of “no” scores 1 point. The OFI-8 score ranges from 0 to 11, with higher scores indicating poorer oral health status. In this study, all participants were divided into two groups: the oral frailty group (OFI-8 ≥ 4) and the non-oral frailty group (OFI-8 < 4).

#### Social isolation

In this cross-sectional survey, the Lubben Social Network Scale (LSNS - 6) was employed to assess social isolation among middle-aged and older adult patients with chronic diseases. The LSNS - 6 scale (41) is primarily designed to evaluate the social networks of middle-aged and older adult individuals. The scale consists of 6 items, encompassing two dimensions: family networks and friend networks. Each item is scored on a scale of 0 to 5, with the total score of the LSNS - 6 scale ranging from 0 to 30. A lower score indicates a higher risk of social isolation, with a total score below 12 indicating the presence of social isolation.

#### Family health

In this cross-sectional study, the family health of participants was assessed using the Chinese version of the Family Health Scale-Short Form (FHS-SF) (42), which is used to measure family health functioning. The FHS-SF consists of 10 items, encompassing four dimensions: family internal emotional communication factors (3 items), family healthy lifestyle factors (2 items), family health resource factors (3 items), and family external social support factors (2 items). Each item is scored on a Likert 5-point scale (1–5). The total score is obtained by summing all items, with the FHS-SF total score ranging from 10 to 50, where a higher score indicates better family health functioning.

#### Quality control

In the cross-sectional study on depressive symptoms among middle-aged and older adult patients with chronic diseases, the research team implemented the following measures to ensure the reliability and validity of the findings. First, during the study design phase, the team clearly defined the inclusion and exclusion criteria for all investigators to ensure sample homogeneity. Second, the study employed a standardized depression assessment tool (PHQ-9). The PHQ-9 scale has undergone cross-cultural validation and demonstrates excellent sensitivity and specificity. Finally, during data collection, the team conducted rigorous investigator training beforehand and adhered to standardized procedures throughout the survey to minimize bias. For example, investigators use standardized instructions to ensure consistent understanding of questionnaire items; all paper-based surveys undergo double verification by investigators during completion to guarantee the authenticity and accuracy of raw data. Ultimately, through the rigorous quality control across these multiple stages, the internal validity and authenticity of this cross-sectional study can be significantly enhanced, providing reliable reference for clinical interventions.

### Data analysis

In this cross-sectional study, the research team conducted data analysis using SPSS 20.0 and Python 3.8. Continuous variables were characterized descriptively, and the Kolmogorov–Smirnov test was employed to verify whether the test data followed a normal distribution. Measurement indicators that did not conform to normal distribution were expressed as M (Q1, Q3). Non-parametric tests were used for within-group or between-group comparisons. Additionally, categorical data were presented as *n* (%), and the chi-square test was applied for comparative analysis of at least two groups, with statistical significance set at *p* < 0.05. The Lasso regression method was employed to select variables, and the variables selected via Lasso regression were utilised in the logistic regression model to determine the final machine learning input features.

### Machine learning model development and visualisation

During the development of the machine learning model, a random seed was set to ensure the reproducibility of results, and a stratified random sampling method was used to split the dataset into a training set and an internal validation set in a 7:3 ratio, for model training and internal validation, respectively. Based on the training set, a total of nine machine learning models were constructed: Logistic Regression (LR), Random Forest (RF), eXtreme Gradient Boosting (XGBoost), Neural Network (nnet), Adaptive Boosting (AdaBoost), Gradient Boosting Machine (GBM), Light Gradient Boosting Machine (LightGBM), K-Nearest Neighbours (KNN) and Support Vector Machine (SVM). Prior to model training, hyperparameter optimisation was performed using grid search combined with 10-fold cross-validation, and the final models were constructed based on the optimal hyperparameters. Subsequently, each model was applied to the training set and internal validation set for prediction. Models were comprehensively evaluated across multiple dimensions—including discriminatory power and overall classification performance—using metrics such as receiver operating characteristic (ROC) curves and area under the curve (AUC), Brier score, recall, F1 score, precision and accuracy. Finally, to better interpret and understand the contribution of each influencing factor in the model to the prediction results, our research team employed the powerful SHAP technique to visualize the best-performing prediction model.

## Results

### Description of the sample

Table 1 presents the demographic characteristics of middle-aged and older adults. Among the 618 middle-aged and older adults, 56.3% (*n =* 348) were male, and the rest were female, 43.7% (*n =* 270). The age range of the middle-aged and older adults was from 45 to 93 years old, with a mean of 67.83 ± 10.550. Place of residence was 239(38.7%), 134(21.7%), and 245 (39.6%) for county, town, and city, respectively. Detailed sociodemographic characteristics of participants are presented in Table 1.

**Table 1:** Presents the detailed baseline characteristics of this cross-sectional study.

Factors		Non-depressed (*N =* 201)	Depressed (*N =* 417)	Statistic	*P-*value
Gender, *n* (%)	Male	99 (28.4)	249 (71.6)	*χ*^2^ = 6.03	0.014
	Female	102 (37.8)	168 (62.2)		
Place of residence, *n* (%)	Rural	56 (23.4)	183 (76.6)	*χ*^2^ = 26.944	<0.001
	Small towns	36 (26.9)	98 (73.1)		
	Cities	109 (44.5)	136 (55.5)		
Are any of your relatives healthcare workers, *n* (%)	Yes	33 (41.3)	47 (58.8)	*χ*^2^ = 3.188	0.074
	No	168 (31.2)	370 (68.8)		
Number of surgeries in the past year, *n* (%)	0 times	117 (38.4)	188 (61.6)	*χ*^2^ = 9.608	0.008
	1 time	36 (25.4)	106 (74.6)		
	≥2 times	48 (28.1)	123 (71.9)		
Hospitalizations in the past two years, *n* (%)	1 time	84 (40.6)	123 (59.4)	*χ*^2^ = 9.203	0.002
	≥2 times	117 (28.5)	294 (71.5)		
Work Status, *n* (%)	Not working	156 (31.3)	343 (68.7)	*χ*^2^ = 1.88	0.17
	Working	45 (37.8)	74 (62.2)		
Marital status, *n* (%)	Unmarried	11 (20.8)	42 (79.2)	*χ*^2^ = 3.659	0.056
	Married	190 (33.6)	375 (66.4)		
Level of education, *n* (%)	No formal education	28 (24.3)	87 (75.7)		
	Elementary school	52 (22.7)	177 (77.3)	*χ*^2^ = 54.55	<0.001
	Junior high school	45 (31.7)	97 (68.3)		
	High school	39 (51.3)	37 (48.7)		
	Vocational school or higher	37 (66.1)	19 (33.9)		
Course of the disease, *n* (%)	<1 year	35 (46.7)	40 (53.3)	*χ*^2^ = 9.298	0.054
	1–3 years	56 (32.9)	114 (67.1)		
	4–5 years	20 (27.4)	53 (72.6)		
	6–10 years	44 (27.8)	114 (72.2)		
	≥11 years	46 (32.4)	96 (67.6)		
Comorbidity, *n* (%)	Yes	150 (30.6)	340 (69.4)	*χ*^2^ = 3.941	0.047
	No	51 (39.8)	77 (60.2)		
Age group, *n* (%)	45–59 years	60 (47.2)	67 (52.8)	*χ*^2^ = 40.401	<0.001
	60–69 years	78 (40.0)	117 (60.0)		
	70–79 years	53 (25.6)	154 (74.4)		
	≥80 years	10 (11.2)	79 (88.8)		
Malignant disease, *n* (%)	Yes	21 (13.8)	131 (86.2)	*χ*^2^ = 32.149	<0.001
	No	180 (38.6)	286 (61.4)		
Social isolation, *n* (%)	Yes	30 (18.6)	131 (81.4)	*χ*^2^ = 19.142	<0.001
	No	171 (37.4)	286 (62.6)		
Oral frailty, *n* (%)	Yes	61 (15.8)	326 (84.2)	*χ*^2^ = 132.551	<0.001
	No	140 (60.6)	91 (39.4)		
Stigma associated with illness, Median (Q1, Q3)		14 (12,17)	24 (20,26)	z = 5.455	<0.001
Family Health, Median (Q1, Q3)		38 (33,41)	30 (26,34)	z = 4.583	<0.001

### Baseline characteristics of participants

**Figure 1:**
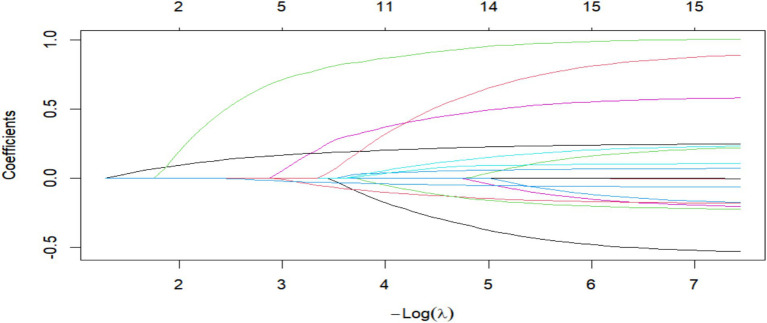
LASSO regression coefficient path diagram.

**Figure 2:**
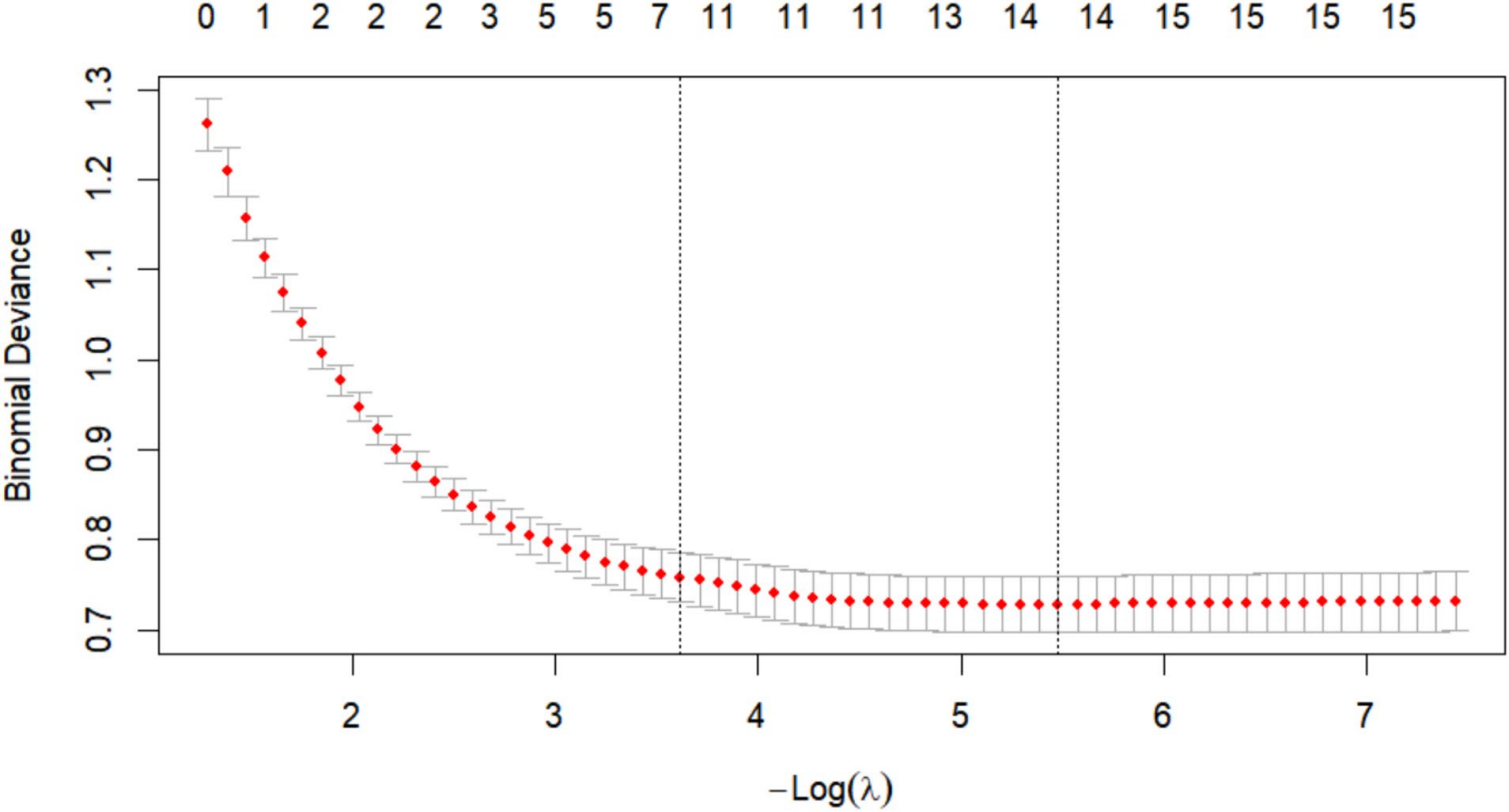
Cross-validation curves for LASSO regression.

In the training set, the presence or absence of depression was used as the dependent variable (yes = 1, no = 0). Lasso regression was employed to identify influencing factors. As shown in Figure 1, the coefficients of the independent variables in the model were gradually reduced, with some coefficients eventually set to zero to avoid overfitting. Figure 2 indicates that, through 10-fold cross-validation, the optimal number of factors was determined to be 11, thereby minimizing the error. Ultimately, 11 influencing factors were identified (Table 2). These 11 factors were incorporated into a binary logistic regression analysis. The results (Tables 1, 3) indicate that “residential treatment within the past 2 years,” “malignant disease,” “Chronic Disease Stigma Scale,” “social isolation,” “oral frailty,” and “family health status” were identified as independent risk factors for depression in patients.

**Table 2:** Types of chronic diseases among participants.

Chronic disease	Frequency (F)	Percent (%)
Hypertension	309	50.0
Cardiovascular disease	118	19.1
Diabetes	217	35.1
Cerebrovascular diseases	104	16.8
Chronic liver disease	114	18.4
Eye diseases	72	11.7
Malignant tumor	117	18.9
Respiratory system diseases	66	10.7
Digestive system diseases	65	10.5
Others	189	30.6

**Table 3:** Binary logistic regression analysis of depression.

Indices	*β*	Wald	*p*-value	OR	95%CI
Hospitalization in the past 2 years	0.542	3.873	0.049	1.719	1.002–2.949
Malignant disease	0.707	4.856	0.028	2.029	1.081–3.806
Stigma scale for chronic	0.237	69.237	0.000	1.268	1.199–1.341
Social isolation	0.867	7.396	0.007	2.380	1.274–4.447
Oral frailty	1.097	19.427	0.000	2.994	1.839–4.876
Family health	−0.060	6.492	0.011	0.942	0.900–0.986
Constant	−3.012	6.867	0.009	0.049	

### A comparison of the performance of nine machine learning predictive models

**Figure 3:**
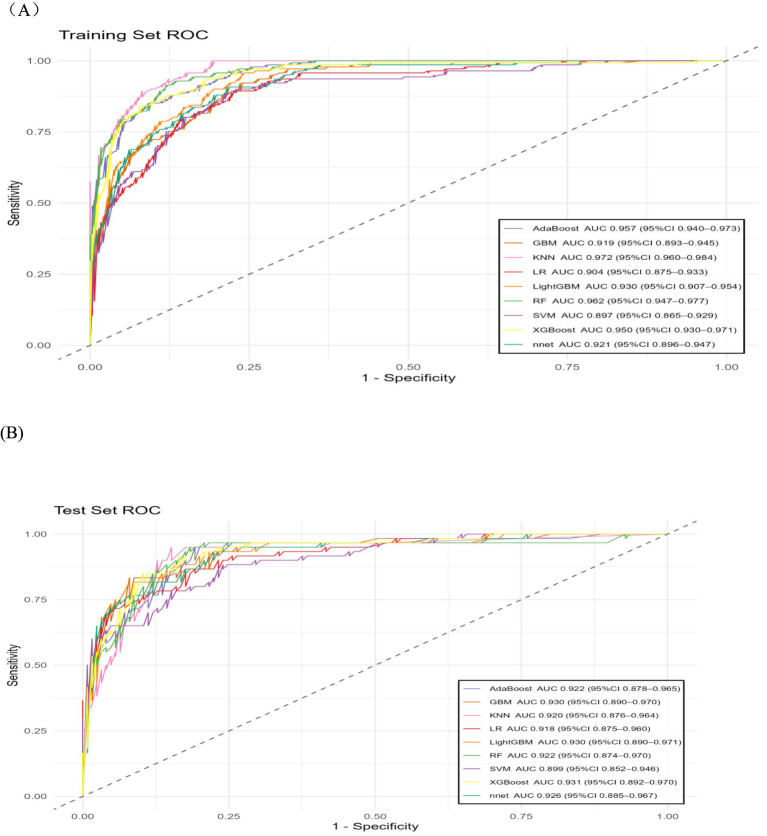
ROC curves for nine machine learning prediction models on the training set and internal validation set.

This study utilized a training set and an internal validation set to evaluate the predictive performance of nine machine learning models—logistic regression (LR), random forest (RF), XG Boost, neural networks (nnet), AdaBoost, GBM, Light GBM, KNN, and SVM—in predicting depression in patients. As shown in Figure 3, the area under the curve (AUC) for all nine models in the training set was above 0.9, with the RF model achieving the highest AUC of 0.962. In the internal validation set, the AUC for all models except the SVM model was above 0.9, with the XG Boost model achieving the highest AUC of 0.931. The XG Boost model achieved an AUC of 0.950 on the training set, demonstrating excellent performance and good stability. Table 4 compares the performance metrics of the nine predictive models. The results indicate that in the internal validation set, the XG Boost model had the highest accuracy (0.881) and F1 score (0.823), as well as the lowest Brier score (0.094). These findings indicate that the XG Boost model demonstrates accurate predictive performance in assessing depression in this specific population. Overall, all models exhibited excellent predictive performance on both the training and validation sets) (Table 5).

**Table 4:** Distribution of influencing factors.

Factors	Assignment
Hospitalization in the past 2 years	1 times = 1, >1 times = 2
Malignant disease	No = 0, yes = 1
Oral frailty	No = 0, yes = 1
Social isolation	No = 0, yes = 1
Stigma scale for chronic	–
Family health	–

**Table 5:** Performance metrics of nine machine learning prediction models on the training set and internal validation set.

Model	Train. accuracy	Train. precision	Train. recall	Train. F1	Train. PPV	Train. NPV	Train. brier	Test. accuracy	Test. precision	Test. recall	Test. F1	Test. PPV	Test. NPV	Test. brier
LR	0.829	0.716	0.748	0.732	0.716	0.884	0.116	0.854	0.75	0.789	0.769	0.75	0.904	0.104
RF	0.896	0.794	0.875	0.833	0.794	0.945	0.078	0.854	0.767	0.78	0.773	0.767	0.896	0.097
SVM	0.818	0.652	0.754	0.7	0.652	0.897	0.116	0.822	0.7	0.737	0.718	0.7	0.88	0.115
XGBoost	0.889	0.823	0.835	0.829	0.823	0.921	0.082	0.881	0.85	0.797	0.823	0.85	0.896	0.094
KNN	0.901	0.809	0.877	0.841	0.809	0.945	0.07	0.843	0.717	0.782	0.748	0.717	0.904	0.106
LightGBM	0.852	0.752	0.785	0.768	0.752	0.901	0.099	0.881	0.8	0.828	0.814	0.8	0.92	0.095
nnet	0.845	0.695	0.803	0.745	0.695	0.918	0.104	0.876	0.75	0.849	0.796	0.75	0.936	0.097
GBM	0.836	0.738	0.754	0.746	0.738	0.884	0.114	0.87	0.767	0.821	0.793	0.767	0.92	0.1
AdaBoost	0.887	0.816	0.833	0.824	0.816	0.921	0.08	0.854	0.85	0.739	0.791	0.85	0.856	0.101

### Building an interpretable predictive model based on the XG boost model

**Figure 4:**
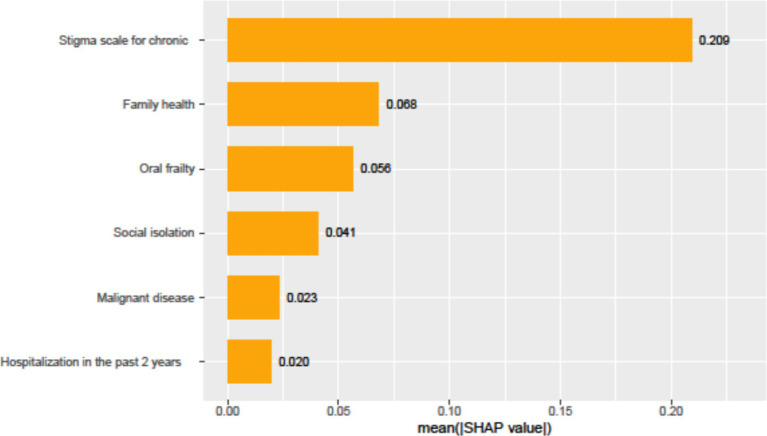
Feature importance ranking diagram.

**Figure 5:**
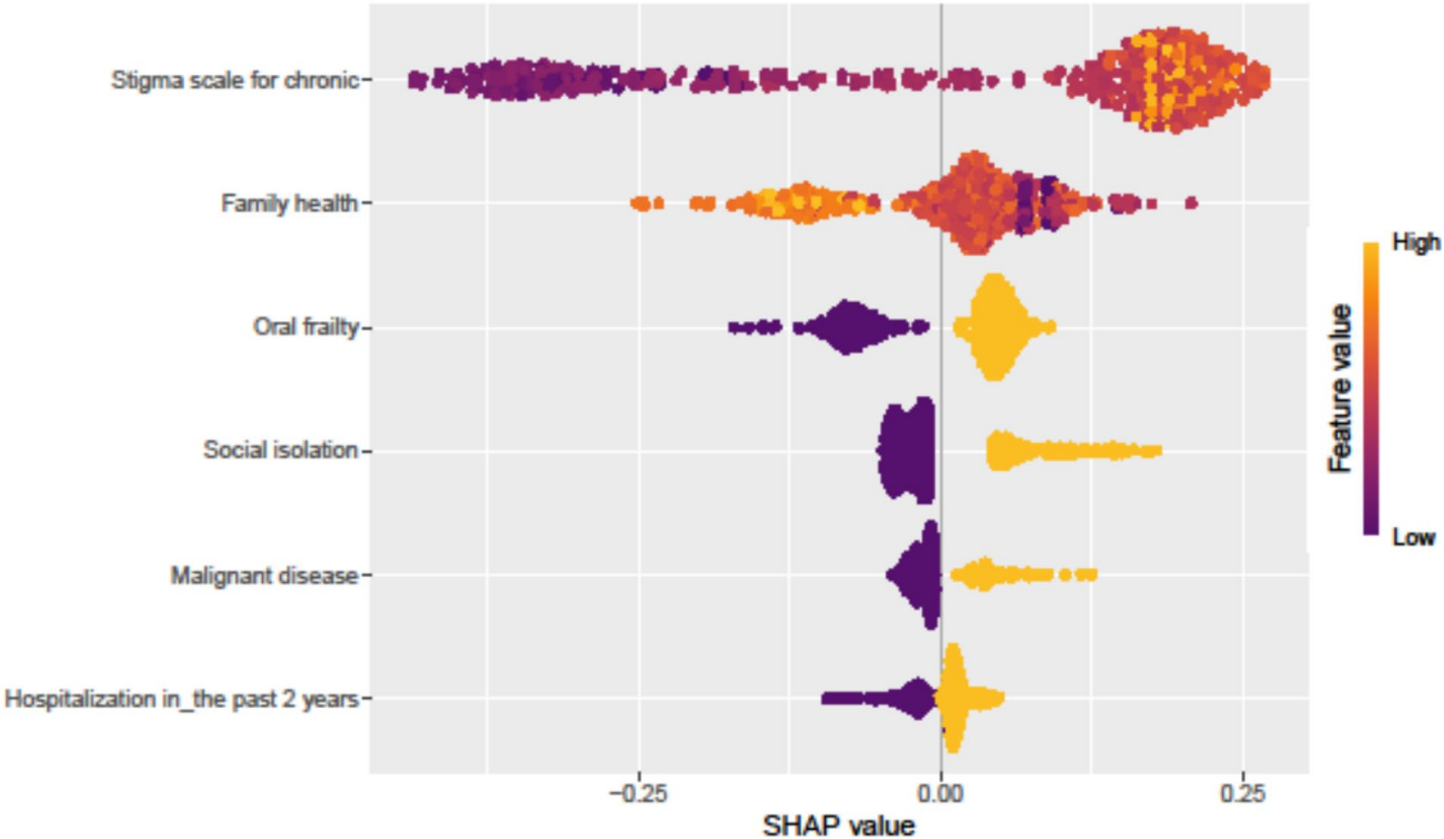
Feature importance swarm plot.

This study utilized an optimized XG Boost model to analyze risk factors for depression in the middle-aged and older adults. SHAP explainability was introduced to address the “black box” problem associated with machine learning models, while also revealing the associations between feature variables and the outcome variable. Figure 4, the feature importance ranking chart, shows that “Stigma scale for chronic,” “Family health,” “Oral frailty,” “Social isolation,” “Malignant disease,” and “Hospitalization in the past 2 years” are ranked in order of their average contribution. The “Stigma scale for chronic” has the highest average contribution at 0.209, indicating a very strong association with depression. Figure 5, the feature importance honeycomb plot, illustrates whether the influence of feature variables on the outcome variable is positive or negative, as well as the distribution of SHA*p* values for each feature. The figure shows that the Stigma Scale for Chronic scores in the study population are concentrated on both sides of the honeycomb plot, indicating a polarized distribution. For a significant portion of the population, SHAP values are skewed toward the right, suggesting a positive association with the onset of depression. Poor family health also has a significant positive impact on the onset of depression, with its SHAP values skewed to the right, indicating that most respondents face numerous family health issues, thereby increasing their psychological burden. Additionally, Social isolation and malignant disease generally exhibit high SHAP values, suggesting that feelings of loneliness and a history of serious illness are also important risk factors. Hospitalization in the past 2 years has a relatively smaller impact but still exerts a positive influence. With very few exceptions, the overall trends across all variables consistently demonstrate a close association between physical and mental health. Social stigma resulting from long-term chronic illnesses, combined with insufficient family support, jointly exacerbate the risk of depression.

## Discussion

In this study, we comprehensively considered the potential factors that have been proposed in previous studies to influence depressive symptoms in middle-aged and older adult patients with chronic diseases and developed a predictive model aimed at predicting the risk of depressive symptoms in middle-aged and older adult patients with chronic diseases. Various validation methods (calibration curves, ROC, and DCA) demonstrated that the model had adequate predictive performance. Our research not only broadens the existing research field on depressive symptoms in middle-aged and older adult hospitalized patients with chronic diseases, but also provides new perspectives for studying the influencing factors of depressive symptoms in this population. Furthermore, it offers references for developing intervention strategies aimed at improving depressive symptoms. In this multicenter cross-sectional study, we found that the prevalence of depressive symptoms (PHQ-9 total score≥10) among middle-aged and older adult chronic disease patients in China was 67.4%, which is higher than the 32.4% reported by Ma et al. (43) in their study of middle-aged and older adult hospitalized patients in Xinjiang. This may be related to factors such as the patient’s long-term suffering from the disease, complex treatment, drug side effects, and social isolation (44). Therefore, providing more social support for the middle-aged and older adults and strengthening community and family interactions are of great significance for improving their mental health (45). Also, regarding the notably high prevalence of depressive symptoms (67.4%) observed in our sample, it is important to consider the specific departmental distribution of the enrolled patients. A substantial proportion of participants were recruited from the interventional department, many of whom were diagnosed with liver tumors. Patients with malignant liver tumors often face a poor prognosis, high symptom burden (e.g., pain, fatigue, ascites), and repeated hospitalizations for interventional procedures such as transarterial chemoembolization or radiofrequency ablation. These factors are known to contribute to severe psychological distress, including hopelessness and depression. Therefore, the overrepresentation of this particularly vulnerable subgroup likely explains, at least in part, the higher prevalence of depressive symptoms compared with previous studies that included general chronic disease populations without a predominance of advanced cancer or pain management cases. Future studies should consider stratifying results by disease severity and department type to further clarify the influence of these factors.

The results of this multicenter cross-sectional study highlight a key public health challenge, which is consistent with global trends observed in similar populations (46). This finding is particularly concerning given the well-documented bidirectional relationship between chronic diseases and depression, where each condition exacerbates the other, leading to poorer clinical outcomes, reduced quality of life, and increased mortality risk (47). The observed prevalence in China exceeds rates reported in other settings, such as 39.8% in Botswana (46), and 24.2% in Brazilian diabetic older adults (47), highlighting the urgent need for targeted mental health interventions in China’s aging population with multimorbidity. According to the findings of this cross-sectional study, patients with more hospitalizations also exhibited higher levels of depressive symptoms. For middle-aged and older adult patients with chronic diseases, increased frequency of hospitalization is often accompanied by disease progression and functional decline, factors that collectively act as risk amplifiers for depressive symptoms. When chronic disease patients experience more negative life events related to their condition during hospitalization, they develop varying degrees of depressive symptoms. Emerging evidence suggests that recurrent hospitalizations not only reflect disease severity but also exacerbate psychological distress, particularly depression, in this vulnerable population. Frequent hospitalizations disrupt daily routines, amplify financial strain, and foster feelings of helplessness—key triggers for depressive episodes (48). As an adverse health outcome, hospitalization may lead to persistent physical pain and activity limitations in patients with chronic diseases. Moreover, the increased frequency of hospitalizations severely restricts individuals’ participation in daily social activities. This may trigger negative emotions and feelings of loneliness, ultimately manifesting as depressive symptoms. Therefore, greater attention should be paid to depression screening and intervention for middle-aged and older adult hospitalized patients with chronic diseases, while providing them with effective disease management and rehabilitation strategies, financial support, and psychological interventions.

In our survey, the depressive symptoms of patients with malignant tumors were more severe than those of patients with other chronic diseases. The severity of depressive symptoms among patients with malignant tumors is notably higher compared to those with other chronic diseases, a finding supported by extensive research on the interplay between chronic illness and mental health (49). Cancer patients often experience a unique psychological burden due to the life-threatening nature of their diagnosis, aggressive treatment regimens, and the pervasive fear of recurrence, which collectively exacerbate depressive symptomatology (50). For instance, studies reveal that 14.9% of middle-aged and older adults with cancer exhibit clinically significant depressive symptoms, a prevalence influenced by factors such as functional limitations, comorbid chronic conditions, and social determinants like marital status and employment (51). In contrast, patients with non-cancer chronic diseases (e.g., diabetes, cardiovascular diseases) demonstrate lower depressive severity, though their mental health remains compromised by disease-related disability and pain (52).

The pathophysiology of depression in malignant tumors is multifaceted, involving biological, psychological, and social dimensions. Cancer-related fatigue (CRF), pain, and inflammation are critical mediators, with hemoglobin levels and C-reactive protein (CRP) serving as biomarkers linking depressive symptoms to disease progression. For example, leukemia patients with subthreshold depression exhibit significantly higher CRF and complication rates, driven by low hemoglobin and elevated CRP, whereas regular exercise emerges as a protective factor (49). Additionally, cancer treatments such as radiotherapy independently amplify depressive symptoms, likely through neuroinflammatory pathways and oxidative stress (53). Notably, persistent depressive symptoms (e.g., CES-D scores >6) are associated with a 1.5-fold increased risk of chronic disease onset in non-cancer populations, but cancer patients face compounded risks due to treatment side effects and psychosocial stressors (54).

This study demonstrates that social isolation is associated with an increased risk of depressive symptoms among middle-aged and older adult patients with chronic diseases, which is consistent with previous research findings (55). Research has found that middle-aged and older adult individuals in a state of social isolation experience a shrinking social network and reduced social participation, with the resulting lack of social connections being a risk factor for depression (56). Middle-aged and older adults with a high degree of social isolation experience reduced social connections, such as decreased contact with their children and fewer organized social activities. When facing negative life events, they often lack communication and emotional support, making them prone to emotional regulation disorders and subsequently increasing the risk of depression (57). Therefore, efforts should be made to strengthen their connections with family and society, providing them with emotional and social support to reduce the risk of depression. Additionally, studies have shown that middle-aged and older adults exhibiting depressive symptoms are more likely to withdraw socially and resist social interactions, which may easily form a vicious cycle between social isolation and mental health problems. Thus, early identification and prevention of social isolation among middle-aged and older adults are crucial (58).

This study found that oral frailty is a risk factor for depressive symptoms in middle-aged and older adult hospitalized patients, and the findings are consistent with previous studies (59). Middle-aged and older adult patients with chronic diseases commonly experience polypharmacy, which increases their risk of drug-induced xerostomia, particularly among antidepressant users (60). It is well recognized that oral frailty is often accompanied by pain and discomfort, negatively impacting individuals’ daily lives, potentially exacerbating depressive symptoms and affecting quality of life (61). Simultaneously, due to poor oral health, middle-aged and older adult individuals have fewer dietary choices compared to others, which may lead to dissatisfaction and contribute to depressive mood (62). Furthermore, issues such as tooth loss can alter facial appearance, affecting self-esteem and causing embarrassment in social situations, thereby reducing social participation. Additionally, dental treatments are often expensive, increasing individual financial burdens and negatively impacting mental health, which may be associated with depressive symptoms (63).

This study found that family health is a protective factor against depressive symptoms in middle-aged and older adult hospitalized patients, which is consistent with previous research findings (26). Family health encompasses social support, family interactions, and resources, all of which are associated with health. These elements contribute to promoting healthier behavioral patterns and reducing disease risks. A supportive family environment fosters a sense of belonging and wellbeing among its members (64), strengthens mutual affection within the family (65), and ultimately benefits social development (66). Family systems theory (67) indicates that among family members, the behavior of an individual member influences the behaviors, cognition, and emotions of other members. When a family member experiences work–family conflict and develops anxiety disorders, providing timely support and assistance can offer positive guidance and constructive feedback to the individual to some extent. A healthy family environment can also provide emotional support, information sharing, and behavioral modeling for patients, enhancing their confidence and ability to manage the disease (68). Meanwhile, family health can also achieve the goal of controlling chronic diseases by improving health literacy and enhancing self-management behaviors (69). The family serves as a crucial environment in an individual’s life, playing a vital role in maintaining mental health. In traditional Chinese culture, the family functions as a “shelter,” with the family system exhibiting unique buffering effects and protective functions for psychological wellbeing (70).

According to the findings of this cross-sectional study, middle-aged and older adult patients with chronic diseases who experienced higher levels of stigma also exhibited more severe depressive symptoms, a result consistent with other research findings. Stigma, viewed as a detrimental psychological issue, can negatively impact patients with chronic illnesses by hindering their adherence to treatment and their ability to manage their conditions effectively (71). This phenomenon results in diminished confidence in the effectiveness of disease management; without timely intervention for these individuals, the challenges associated with treating chronic conditions may escalate, severely compromising their overall quality of life (72, 73). The intersection of stigma and depressive symptoms in patients with chronic diseases represents a critical yet underexplored area in public health and clinical research. Chronic illnesses often carry societal stigma, which exacerbates psychological distress and undermines treatment adherence, creating a vicious cycle that worsens both mental and physical health outcomes (74). Stigma operates through multiple pathways to influence depressive symptoms in chronic disease populations. Perceived discrimination, a form of enacted stigma, has been shown to amplify daily stress reactivity, with heightened threat appraisals and negative affective responses mediating its long-term effects on mental health (75).

Based on the predictive factors from our XGBoost model (chronic illness stigma and oral frailty), we propose a stratified risk-based intervention framework for clinical practice. For high-risk patients (e.g., predicted probability >60%), those with high stigma should receive a brief cognitive behavioral intervention (4–6 individual sessions during hospitalization focusing on cognitive restructuring and coping skills), while those with oral frailty require an integrated inpatient oral health management program (daily mouth care, artificial saliva, dental referral if needed). This predictive model can be embedded into electronic health records as an automated screening tool at hospital admission, flagging high-risk patients on nursing dashboards with minimal extra workload. For high-risk patients, we recommend weekly in-hospital reassessments using the PHQ-9, followed by telephone follow-ups at 2 weeks post-discharge and monthly for 3 months. Regarding cost-effectiveness, the proposed low-intensity, task-shifting model (using trained nurses for both CBI and oral care) incurs modest training and time costs, which are likely offset by reduced antidepressant use, shorter hospital stays, and fewer readmissions – making it feasible for routine implementation in Chinese tertiary hospitals.

Compared with recent machine learning studies on depressive symptoms in Chinese older adults that used community-based cohorts (30–35), our study offers several unique contributions. First, unlike those community-dwelling samples with lower depression rates, we focused specifically on hospitalized patients with chronic diseases, whose prevalence of depressive symptoms reached 67.4% partly due to the inclusion of liver tumor patients from the interventional department. This highlights the need for prediction tools tailored to high-burden inpatient settings. Second, we incorporated novel predictors not available in national surveys – namely oral frailty and a disease-related stigma scale – which showed meaningful feature importance in our model and are particularly relevant for hospitalized populations. Thus, while prior work has demonstrated the feasibility of machine learning for community-dwelling older adults, our study provides the first inpatient-specific model for chronic disease patients that integrates oral frailty and stigma, thereby filling a distinct gap in the literature.

### Limitations

Given the rapid aging of the Chinese population, the issue of depressive symptoms among middle-aged and older adult patients with chronic diseases is becoming increasingly critical. The current research findings were significant for understanding the mechanisms linking oral frailty, clinical physiological resilience, and depressive symptoms, and provide preliminary evidence for implementing intervention strategies for depressive symptoms in the middle-aged and older adult chronic disease patient population. Our study mentions several limitations; first, the cross-sectional design hinders the examination of causal relationships between oral frailty, clinical physiological resilience, and depressive symptoms, necessitating longitudinal designs in the future to confirm these associations. Second, all survey data were self-reported by participants, which may lead to information bias. Third, the samples were collected from a city in Anhui Province, China, so the research findings may not be generalizable to middle-aged and older adult patients with chronic diseases in other countries and regions. Fourth, several important confounding variables that are known to influence depressive symptoms in middle-aged and older adult hospitalized patients with chronic diseases were not controlled for in this study. Specifically, we did not collect data on household income, medical insurance coverage, caregiver burden (e.g., presence, type, and hours of caregiving), or duration of the current hospitalization. These factors have been consistently associated with depression risk in chronically ill populations. For example, lower household income and inadequate insurance coverage may increase financial stress and treatment non-adherence, while high caregiver burden and prolonged hospital stays can exacerbate feelings of helplessness and social isolation. The absence of these variables in our model may lead to residual confounding and could affect the generalizability of our findings. Future studies should incorporate these variables to develop more comprehensive and accurate prediction models for depressive symptoms in this vulnerable inpatient population.

## Conclusion

This study examined the current status of depressive symptoms and their influencing factors among middle-aged and older adult hospitalized patients with chronic diseases. Overall, the study confirmed previous hypotheses. The findings revealed that number of hospitalizations, malignant diseases, social isolation, and oral frailty were significantly associated with depressive symptoms, while family health served as a protective factor against depressive symptoms in middle-aged and older adult hospitalized patients with chronic conditions. Reducing the prevalence of depressive symptoms is crucial for improving the physical function and quality of life of middle-aged and older adult patients with chronic diseases, as well as for decreasing the occurrence of adverse health outcomes. It is recommended that we comprehensively assess middle-aged and older adult in patients with chronic diseases and develop multimodal, personalized mental health interventions.

## Data Availability

The raw data supporting the conclusions of this article will be made available by the authors, without undue reservation.
